# Nutritional, physicochemical and sensorial acceptance of functional cookies enriched with xiquexique (*Pilosocereus gounellei*) flour

**DOI:** 10.1371/journal.pone.0255287

**Published:** 2021-08-10

**Authors:** Tamires Alcântara Dourado Gomes Machado, Maria Teresa Bertoldo Pacheco, Rita de Cássia Ramos do Egypto Queiroga, Letícia Medeiros Cavalcante, Fabrícia França Bezerril, Rita de Cássia Salvucci Celeste Ormenese, Aline de Oliveira Garcia, Elizabeth Harumi Nabeshima, Maria Manuela Estevez Pintado, Maria Elieidy Gomes de Oliveira

**Affiliations:** 1 Pós-graduação em Ciência e Tecnologia de Alimentos, Centro de Tecnologia de Alimentos, Universidade Federal da Paraíba, João Pessoa, Paraíba, Brazil; 2 Centro de Química de Alimentos e Nutrição Aplicada, Instituto de Tecnologia de Alimentos, Campinas, São Paulo, Brazil; 3 Departamento de Nutrição, Centro de Ciências da Saúde, Universidade Federal da Paraíba, João Pessoa, Paraíba, Brazil; 4 Centro de Biotecnologia e Química Fina, Escola Superior de Biotecnologia, Universidade Católica do Porto, Porto, Portugal; Indian Institute of Food Processing Technology (IIFPT), INDIA

## Abstract

The objective of this study was the production of innovative functional cookies enriched with two different sizes (100 and 28 mesh) xiquexique flour by substitution ratio 50% of wheat flour and monitoring the impact of these enrichments on the nutritional, physicochemical, texture characteristics and consumer acceptance. The physicochemical characteristics and sensorial properties of the xiquexique cookies were evaluated in a pursuit to identify an innovative bakery ingredient with high nutritional value and potential function that could be exploited by the food industry. The water activity and moisture values were low, which can provide greater stability during storage of food matrices, such as cookies. The xiquexique cookies had greater ash (2.47–2.74%), protein (0.94–1.36%), fiber (4.41–8.10%), and resistant starch (3.65–2.10%) contents than their respective controls with 100% wheat flour. The functional cookies were rich in minerals: mainly calcium, iron, potassium, magnesium and manganese and can be consumed by all individuals to help meet daily needs, especially those of people who have increased needs for these essential nutrients. In addition to the darker color of the xiquexique cookies, the hardness of these was higher than that of the control cookies, while the expansion index was smaller. The data from the Check All That Apply sensory method, which consists of a test used mainly for recipe adjustments and the development of ideal food products, confirmed that xiquexique flour have the potential for the development of bakery products such as cookies.

## Introduction

Unconventional plant species have gained global attention for use in the food industry because they are nutritive and particularly rich in bioactive compounds. Several studies have observed the potential of these species for the development of functional food products [1–3]. Unconventional food plants (UFP) were defined as edible species that have one or more parts with food potential and no common use, including native and exotic plants and cultivated and spontaneous plants [4].

As an example of UFP we have *Pilosocereus gounellei* which is a cactus in the Cactaceae family that grows in the semiarid region of Brazil and multiplies regularly, covering extensive areas of the Caatinga; moreover, this plant is popularly known as xiquexique [5]. The cladodes, which are modified stems, are usually eaten peeled, fresh or cooked, and can also be used in the production of cakes, sweets, cookies, flours [6–8] and jam [9].

This UFP (*in natura* cooked or dry) can be an alternative crop with multiple uses and benefits, providing human beings with excellent nutritional characteristics, as they are rich in minerals, carbohydrates, and fibers and low in fat and sugars [10]. In addition, this species has an antioxidant capacity due to the presence of phenolic compounds [11], anti-inflammatory activity [12], and gastroprotective effects [13]. Thus, xiquexique can be an interesting ingredient in the development of breakfast cereals, fruit juices, bakery and meat products, sauces, shredded cheeses, cookies, pastas, snacks, frozen desserts, and many other food products.

Xiquexique central stems (cladodes without the peel and pulp) are suitable for the production of flour, while the pulp is more appropriate for the preparation of products such as jam and candies [9, 10]. The key advantages of using xiquexique flour are their mineral, fiber and resistant starch contents, which may encourage its application in various dry food items and the preparation of bakery products and beverages. Another advantage of using xiquexique flour in the processing of food products is the cost of its production. According to FAO’s Investment Centre Division [14] the average cost for annual wheat flour production would be USD 140.1/ton of wheat grains; while the cost for the annual production of xiquexique flour would be around USD 78.14/ton of xiquexique cladode, considering the fixed and variable costs of a technological process of a self-sustainable food matrix.

New technologies and ingredients are being introduced worldwide to fulfill nutritional needs [15, 16]. Functional foods, including whole foods and fortified, enriched, or enhanced foods or dietary components, may reduce the risk of chronic disease and provide health benefits beyond basic nutrition. Screening novel fiber sources will be the next challenge to significantly impact gut microbiota-associated human diseases [17]. The fibers consumption has preventive effects that likely reflect biological activity and show anticancerous, antibacterial, anti-inflammatory, antioxidative and antiapoptotic effects [18]. Due to so many advantages, the addition of fiber-containing ingredients in food matrices such as cookies is pertinent.

Cookies occupy an important position in the snack food industry due to their variety in taste, crispiness and digestibility, consequently, they represent a valuable vehicle of supplementation with nutrients because of their popularity, relatively low cost, varied taste, ease of availability, high nutrient density and long shelf life [19]. Cookies are made via a wide variety of styles using an array of ingredients, including chocolate, butter, peanut butter, nuts or dried frosting [20]. Wheat flour is the principal component of virtually all cookies; however, good quality products can be prepared using nonwheat (nongluten) flours [21], such as xiquexique flour. When processing a food product, such as a cookies, understanding the functions of ingredient and process variables is necessary for the industrial production of fiber-fortified cookies to attain optimum product quality [22].

To date, few studies have focused on the physicochemical parameters, mineral content and antioxidant potential of natural xiquexique [23]. However, to our knowledge, there is still no scientific data that reports the development and nutritional composition of xiquexique cookies. This study aimed was the production of innovative functional cookies enriched with two different mesh sizes xiquexique flour by substitution ratio 50% of wheat flour and monitoring the impact of these enrichments on the nutritional, physicochemical, texture characteristics and consumer acceptance.

## Materials and methods

### Materials

Cladodes of *P*. *gounellei* plants were collected in Boa Vista (PB), Brazil, in November 2016. Botanical identification was conducted by Prof. Dr. Leonardo Felix Person (DF/CCA/UFPB), and a voucher specimen was deposited in the Professor Jaime Coelho de Morais—Center for Agricultural Sciences Herbarium, Federal University of Paraíba (CCA/UFPB) under code 17.562.

**Fig 1:**
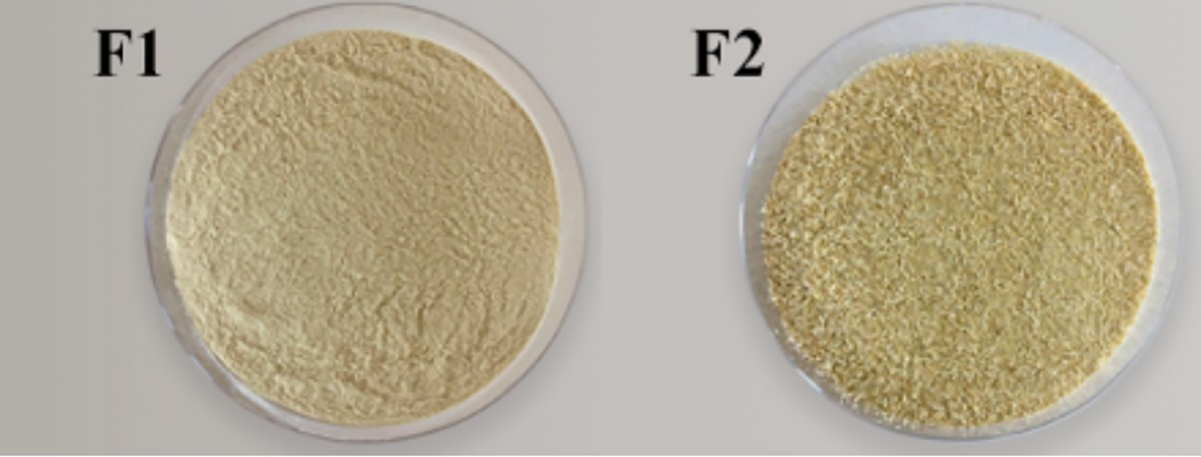
Xiquexique flour. Xiquexique flour tamized at 100 mesh (F1) and xiquexique flour tamized at 28 mesh (F2).

To obtain the xiquexique flour (tamized at 100 and 28 mesh), the collected material (cladodes) was carefully sanitized with running water and sodium hypochlorite (100 ppm/15 min) for soil removal and decontamination. The central stem was stripped, and the pulp and peel were removed. Then, the central stems were cut into 1 cm slices, and their pieces were autoclaved (121 ±1°C/20 min). Subsequently, samples were cooled at room temperature, followed by drying in an air circulation oven (40 ±1°C) until reaching approximately 4% moisture. After drying, the xiquexique was ground in a knife mill (Willey, Solab^®^, Piracicaba, São Paulo) and screened with a 100- and 28-mesh sieve on a sieve shaker, resulting in two samples, flour 1 and flour 2 (F1 and F2), which are shown in Fig 1. The flour was vacuum-sealed in sterile polyethylene bags at approximately 100 g per bag, rolled in aluminum foil and frozen (-20 ±1°C) until cookies production. The remaining cookies ingredients: crystal and brown sugars, natural vanilla bean, eggs, drops of milk chocolate, chopped nuts (0.5 cm), wheat flour, oats flakes (0.7 x 0.1 cm), unsalted butter and salt were obtained from a local market in Campinas, SP, Brazil.

### Preparation of xiquexique cookies

**Fig 2:**
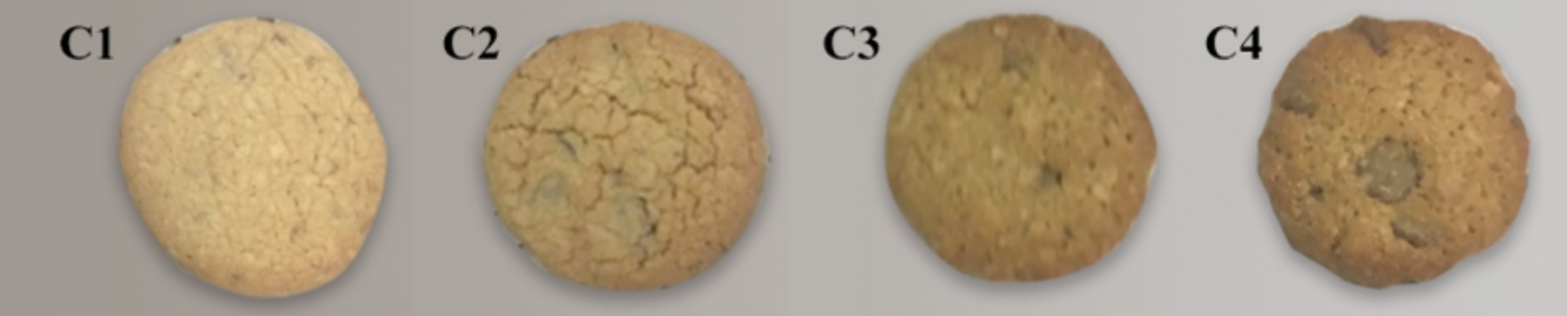
Xiquexique cookies. C1—wheat flour cookies (control1), C2–50% xiquexique flour (100 mesh) cookies, C3—whole wheat flour cookies (control2) and C4–50% whole xiquexique flour (28 mesh) cookies.

Some recipes of conventional and whole cookies were tested to determine the formulations to use. In order to achieve a standardized processing method for control formula, a preliminary experiment was held and two control formula out of 5 tested formulas were chosen based on sensorial characteristics. The cookies ingredients and their proportions are shown in Table 1. Four formulations were produced (Fig 2): C1—wheat flour cookies (control1), C2–50% xiquexique flour (100 mesh) cookies, C3—whole wheat flour cookies (control2) and C4–50% whole xiquexique flour (28 mesh) cookies. From the control formulation, cookies were produced by applying different levels of substitution of wheat flour with the finely ground xiquexique flour (100 mesh) and the coarsely ground xiquexique flour (28 mesh). The percentage of this substitution of wheat flour was 50% to improve nutritional value and observe the influence of addition this flour on cookies.

**Table 1:** Cookies formulations.

Ingredients (g)	C1	C2	C3	C4
Wheat flour	420	210	280	140
F1	-	210	-	-
F2	-	-	-	140
Butter	264	264	200	200
Crystal sugar	180	180	180	180
Brown sugar	180	180	180	180
Egg	183	183	359	359
Chocolate	300	300	300	300
Walnuts	400	400	400	400
Vanilla	0.12	0.12	0.12	0.12
Salt	4	4	4	4
Yeast	8	8	8	8
Oat flakes	-	-	180	180

Description of cookies: C1—wheat flour cookies (control1), C2–50% xiquexique flour (100 mesh) cookies, C3—whole wheat flour cookies (control2) and C4–50% whole xiquexique flour (28 mesh) cookies. Description of the xiquexique flour: xiquexique flour tamized at 100 mesh-F1, xiquexique flour tamized at 28 mesh-F2.

Cookies processing was performed using a methodology adapted from Kaur et al. [2]. The ingredients were weighed, and the fluid ingredients were homogenized for 60 sec in a stand mixer (Kitchen Aid^®^, model K5SS) at medium speed and equipment level 4 (144 rpm) with a flat beater attachment. The sugars (crystal and brown) were added and mixed for 3 min at medium speed. Then, the remaining dry ingredients were added and homogenized for 1.5 min at low speed, level 2 (96 rpm), until complete homogenization.

The dough was rolled out on a table with a Teflon rolling pin to a thickness of 8 mm by use of a guide. Cookies were cut with a 3.5 mm (diameter) aluminum mold, placed on a perforated metal tray, and baked for 9 min in a Vipinho 0448 oven (Perfecta^®^, Curitiba, Paraná) preheated to 180°C. The cookies were packed in paper packages and then in laminate bags, sealed under vacuum and stored at room temperature (25°C) until analysis (1 day).

### Physicochemical and nutritional characteristics

Physicochemical and nutritional characteristics analyses were conducted in triplicate according to standard procedures [24, 25]: Water activity (a_w_) and moisture, ash, lipid, protein, soluble, insoluble e total fiber, starch and resistant starch contents were determined. The a_w_ at 25°C was determined using an Aqualab^®^ apparatus (model C2-2 Water Activity System^®^, Washington-USA) in accordance with the manufacturer’s instructions. The soluble, insoluble, and total dietary fiber contents were determined using Megazyme K-TDFR kit (Megazyme International Ireland Ltd, Bray, Ireland) and following the instructions of the kit which based on an integrated enzymatic-gravimetric analysis procedure [24]. The moisture and total solid contents were determined by drying, the ash content was quantified by carbonization followed by incineration in a muffle furnace (550°C), the lipid content was determined based on the Soxhlet method, and the protein content was quantified using the Micro-Kjeldahl method [24]. The determination of the starch content was performed by polarimetry [25] and resistant starch was determined by a Megazyme resistant starch assay kit (K-RSTAR, Wicklow, Ireland) [24]. Resistant starch was calculated as the amount of glucose multiply by 0.9.

### Mineral profile

Mineral content analysis was performed in triplicate on an inductively coupled plasma atomic emission spectrometry (ICP-OES 5100 VDV, Agilent Technologies^®^, Tokyo, Japan) instrument. The analytical conditions were as follows: radiofrequency power, 1.2 kW; plasma flow, 12 L/min; auxiliary flow rate, 1.0 L/min; nebulization flow, 0.7 L/min; plasma vision, axial to Fe, Mn, Se and Zn and radial to Ca, Mg, P, and Na; Fe, 259.553 nm; Mn, 257.610 nm; Se, 196.026 nm; Zn, 206.2200 nm; Ca, 317.933 nm; Mg, 279.553 nm; P, 213.618 nm; and Na, 589.592 nm.

### Color, texture and spread ratio

The instrumental color was measured in triplicate using a CR-400 colorimeter (Minolta Co., Osaka, Japan). The CIE Lab color scale (L*a*b*) was used with a D65 illuminant (standard daylight) and a 10° observer angle. The L*, a* and b* parameters were determined by values reported for the lightness, L* (L* = 100 for white; L* = 0 for black), chroma, a* (+ a* for redness;—a* for greenness), and hue, b* (+ b* for yellow;—b* for blueness,) parameters of the CIELAB system were the mean of three measurements at three different locations on each cookies sample.

The texture was determined using a texture analyzer (model TA-XT2i, Stable Micro systems, U.K.) to measure the hardness (as fracture force) of cookies via a 3-point bending test using a 3-point bending rig, a trigger force of 25 g, and a load cell of 50 kg. The textural studies were conducted at a pretest speed of 1.5 mm/s, a test speed of 2.0 mm/sec, a posttest speed of 10 mm/sec, and a distance of 10 mm, and the distance between the two bottom supports was adjusted to 50 mm. The peak value was measured as fracture force was recorded at a point when the cookies were broken into two major pieces and reported as hardness [26]. The peak force (PF, g) at the breaking point represented the breaking strength of the cookies. Mean values of triplicate analyses are reported as the fracture force. The PF difference (ΔE) of the without (control) or with xiquexique (test) to conventional and whole cookies were calculated using the equation below, where the PF0 = control and PF1 = test.


$$\Delta E=\sqrt{(PF0-PF1{)}^{2}}$$ (1)

The spread ratio was determined according to AACC [27] as the ratio between the average length of 8 aligned cookies (diameter) and the average value of the cookies height (thickness).

### Sensory properties

Evaluation of microbiological parameters (*Salmonella*/25g, Presumptive *B*. *cereus*/g, *Escherichia coli*/g, Molds and yeasts/g) to determine the security for consumption followed the methodology recommended by APHA [28] and showed that the cookies samples were not contaminated and therefore able to be consumed in sensory tests. Declare that the present study was carried out in accordance with the Research Ethics Committee of the Health Sciences Center of the Federal University of Paraiba—CEP/CSS. The approval number is CAAE: 79748617.2.0000.5188, and the form of consent was obtained written. Informed written consent was obtained from willing participants before the start of the study.

The sensorial evaluation of the developed products was carried out 1 day after their manufacture by 123 consumers using acceptance tests [29]. The effects of hardness intensity on biting and crunching and sweetness regarding the ideal overall acceptability were evaluated [30], and descriptive analysis was carried out by the Check All That Apply (CATA) method [31]; moreover, consumption intentions were recorded [29].

Consumers aged between 18 and 60 years old were recruited from ABC social classes [32]; pregnant women and children were not they included. The products were evaluated for acceptability of the appearance, color, aroma, crunchiness, flavor, and overall acceptability parameters through a structured hedonic scale of nine points (9 = liked extremely, 5 = neither liked nor disliked and 1 = disliked extremely). The samples were also evaluated for the effect of texture on biting and crunching and sweetness employing the ideal 5-point scale (5 = much harder/crunchier/sweet than I like, 3 = the way I like it, 1 = much less hard/crunchy/sweet than I like).

For the CATA descriptive analysis, a list of 15 descriptors was presented, and consumers were asked to choose descriptors from this list that were present and characterized the product being evaluated and general perception was conducted by penalty analysis.

Consumers were then asked about the intended consumption of the samples on a 7-point scale (7 = I would consume this product very often, 4 = I would consume this product when available, but I would not go out of my way, 1 = I would consume this product if I had no other choice).

The tests were performed in individual booths using white light, away from noises and odors, and equipped with Compusense Five version 5.6 for data collection and analysis.

### Statistical analyses

The data were submitted to analysis of variance (ANOVA) followed by Student’s t-test to verify significant difference between the samples, considering p < 0.05, and using XLSTAT software version 2019.1.2 (Addinsoft 2019; XLSTAT statistical and data analysis solution, Boston, MA, USA) [33].

## Results and discussion

### Physicochemical and nutritional characteristics

Physicochemical and nutritional characteristics analyses were conducted to examine the composition and nutritional value of the xiquexique cookies, and the results are summarized in Table 2. The substitution of wheat flour with xiquexique flour (tamized at 100 and 28 mesh) influenced all cookies parameters (p < 0.05), except for the lipid content (p ≥ 0.05). The conventional and whole cookies exhibited similar a_w_ values and moisture contents, and the inclusion of xiquexique flour in xiquexique cookies (C2 and C4) contributed to a reduction in these values when compared to their control cookies (C1 and C3, respectively), which indicates that these flours (F1 and F2) will be stable during storage.

**Table 2:** Physicochemical properties of the functional cookies.

Variable	C1	C2	C3	C4
a_w_	0.374^a^ ±0.010	0.319^b^ ±0.010	0.590^a^ ±0.010	0.430^b^ ±0.010
Moisture %	3.47^a^ ±0.02	2.63^b^ ±0.06	3.60^a^ ±0.01	3.50^b^ ±0.01
Ash %	1.33^b^ ±0.02	2.47^a^ ±0.02	1.55^b^ ±0.01	2.74^a^ ±0.02
Protein %	0.82^b^ ±0.01	0.94^a^ ±0.01	1.25^b^ ±0.01	1.36^a^ ±0.01
Lipids %	24.08 ^a^ ±0.99	25.52 ^a^ ±0.17	28.46 ^a^ ±0.87	29.33 ^a^ ±0.10
Total dietary fibers %^1^	2.13^b^ ±0.04	4.41^a^ ±0.02	3.38^b^ ±0.06	8.10^a^ ±0.01
Insoluble fibers %	1.03^b^ ±0.04	4.33^a^ ±0.04	2.43^b^ ± 0.04	7.03^a^ ±0.01
Soluble fibers %	1.10^a^ ±0.07	0.08^b^ ±0.01	0.95^b^ ±0.01	1.07^a^ ±0.01
Starch %	20.80^a^ ±0.04	20.63^b^ ±0.01	17.65^a^ ±0.01	16.94^b^ ±0.10
Resistant starch %	1.81^b^ ±0.05	3.65^a^ ±0.01	1.32^b^ ±0.12	2.10^a^ ±0.19

C1—wheat flour cookies (control1), C2–50% xiquexique flour (100 mesh) cookies, C3—whole wheat flour cookies (control2) and C4–50% whole xiquexique flour (28 mesh) cookies.

^a-b^Mean ± standard deviation with different letters on the same row differed significantly by Student’s t-test (p < 0.05) for C1 versus C2 and C3 versus C4.

^1^Daily recommended fiber intake for men (19–50 years old), women (19–50 years old) and children (1–3 years old) is 38, 25 and 19 g, respectively. Considering a 50 g portion, supply (%) C1 (2.80, 4.26, 5.61), C2 (5.80, 8.80, 11.60), C3 (4.45, 6.76, 8.89) and C4 (10.66, 16.20, 21.32) for men, women and children, respectively. Based of Institute of Medicine. Dietary Reference Intakes, Washington D. C., National Academy Press; 2003 (1997–2005) [34].

The ash content of both xiquexique cookies (C2 and C4) was greater than that of their respective controls (C1 and C3), probably due to the xiquexique flour (tamized at 100 and 28 mesh) being a rich source of minerals (S2 Table). The protein contents of C2 and C4 were greater than those of C1 and C3 (p < 0.05), but it did not show an expressive increase as in results were reported by Bouazizi et al. [35] when evaluating the protein content of the prickly pear (*Opuntia ficus-indica* L.) cookies.

Concerning the fiber content, the substitution of wheat flour with the xiquexique flour with two different particle sizes (100 and 28 mesh), contributed to an increase in the total and insoluble fiber contents, in addition to reducing the content of soluble fibers in the xiquexique cookies C2 (p < 0.05). This could have been because of the granulometry difference; the 100-mesh flour had lower soluble fiber content than that of the 28-mesh flour (S1 Table).

In the literature it was seen that the addition of cactaceous flours or substitution of wheat flour for cacti flours contributed to the increase of total and insoluble dietary fibers in cookies. This was the case in the study by Bouazizi et al. [35], who worked with replacing up to 30% of wheat flour with prickly pear shell flour (*Opuntia ficus-indica* L.) in the preparation of cookies. Dick et al. [36] working with mucilage and cactus cladode flour (*Opuntia monacantha*) as alternative ingredients in gluten-free cookies observed that the addition of 10 and 15% of this flour contributed to the increase in the total and insoluble dietary fiber content. Equal behavior about fibers was also observed in our study with the replacement of up to 50% of wheat flour by xiquexique flour sifted in 100 or 20 mesh. However, the results in our study for total and insoluble fibers exceeded the values detected in the above-mentioned surveys.

Fibers can be considered key compounds that preserve the gut microbiota because they can be fermented by the microbiota, producing metabolites that work to improve consumer health [17]. In this way, reference values for their intake have been periodically established [31] considering dietary reference recommendations (RDAs) and adequate intakes (AIs). These parameters determine the levels of nutrients corresponding to the needs of an individual [34]. The daily recommended fiber intake for men (19–50 years old), women (19–50 years old) and children (1–3 years old) is 38, 25 and 19 g, respectively.

Considering a 50 g portion (3 cookies with approximately 16.6 g each), according to recommendations of the dietary reference intake (DRI), C2 and C4 would be responsible for supplying 5.80 and 10.66% of the total fiber DRI, respectively, while control cookies (C1 and C3) supplied just 2.80 and 4.45% of the DRI for men (19 to 50 years old).

The recommendations for women (19 to 50 years old) and children (1 to 3 years old), these values were higher, providing 8.82 (C2) and 16.20% (C4) of the DRI for women and 11.61 (C2) and 21.32% (C4) of the DRI for children, while control samples provided just 4.26 (C2) and 6.76% (C4) of the DRI for women and 5.61 (C2) and 8.89% (C4) of the DRI for children. These control cookies values corresponded to less than 50% of fiber supplied by the xiquexique cookies.

The starch content of xiquexique cookies was lower than that of control cookies, while the resistant starch content was higher (p < 0.05). This could be justified by the low starch content and high resistant starch content determined in the xiquexique flour (F1 and F2) used, respectively, in the processing of C2 and C4 cookies (S1 Table).

Principal carbohydrate of all cereals is starch, and wheat cookies present an average of 42% starch [21]. Starch composition is an important parameter because of the nutritional and technological characteristics of food product applications, such as texture and expansion index [37]. Thus, the xiquexique cookies (C2 and C4) obtained lower starch contents and higher resistant starch contents than the control cookies (C1 and C3), considering that the nutritional and technological features were consistent with control cookies. A study with alfalfa seed flour in cookies showed similar results to ours for total starch and resistant starch, with a decrease in total starch and an increase in resistant starch in alfalfa cookies compared with that in the controls [38].

In a study by Zamora-Gasga et al. [39] with granola bars prepared with *Agave tequilana* ingredients, it was found that the replacement of whole wheat flour by agave dietary fiber contributed to the reduction of starch content and increase in the concentration of total and insoluble dietary fibers in the experimental bars. Similar behavior was also observed in our research, in which it was found that the substitution of 50% of wheat flour (refined or whole) by xiquexique flour with granulometry of 100 or 28 mesh resulted in cookies with lower starch contents and larger quantities of total and insoluble dietary fibers in their constitution.

### Mineral profile

The mineral profiles of xiquexique cookies are presented in Table 3. According to S2 Table, F1 and F2 xiquexique flours used, respectively, in the processing of cookies C2 and C4 had a high concentration of minerals, which contributed to these formulations having higher levels for most detected minerals (p < 0.05), when compared to their respective control cookies—C1 and C3 (without adding xiquexique flour).

**Table 3:** Minerals profile in mg/100 g of cookies.

Elements	C1	C2	C3	C4	*Recommendation (mg)
K	212.43^b^ ±1.48	508.92^a^ ±2.68	264.57^b^ ±3.53	555.77^a^ ±1.93	4700^(^^1)^
Ca	90.39^b^ ±1.16	201.45^a^ ±3.15	98.63^b^ ±0.89	124.88^a^ ±0.45	1000^(^^1)^
P	172.59^a^ ±2.14	151.97^b^ ±1.18	246.50^a^ ±0.82	237.81^b^ ±0.88	700^(2)^
Mg	44.18^b^ ±1.04	151.08^a^ ±1.06	65.14^b^ ±1.53	132.88^a^ ±2.12	420^(2)^
Na	183.09^b^ ±1.50	222.94^a^ ±1.97	173.95^b^ ±3.01	218.36^a^ ±2.98	1500^(^^1)^
Cu	0.24^a^ ±0.01	0.25^a^ ±0.01	0.44^a^ ±0.01	0.42^a^ ±0.01	0.9^(2)^
Fe	2.54^b^ ±0.01	2.80^a^ ±0.08	3.19^a^ ±0.01	2.87^b^ ±0.02	8^(^^1)^
Mn	0.94^b^ ±0.03	7.23^a^ ±0.02	1.69^b^ ±0.08	5.36^a^ ±0.17	2.3^(^^1)^
Zn	0.94^a^ ±0.02	0.90^b^ ±0.01	1.59^a^ ±0.03	1.48^b^ ±0.01	11^(2)^

C1—wheat flour cookies (control1), C2–50% xiquexique flour (100 mesh) cookies, C3—whole wheat flour cookies (control2) and C4–50% whole xiquexique flour (28 mesh) cookies.

^a, b^Media ± standard deviation with different letters on the same row differed significantly by Student’s t-test (p < 0.05), for C1 versus C2 and C3 versus C4.

*Baseado em Institute of Medicine. Dietary Reference Intakes, Washington D. C., National Academy Press; 2003 (1997–2005). Based on a 70 kg man, 31–50 years old [34].

(1) Adequate Intake

(2) Recommended Dietary Allowances.

The results presented relevant amounts of minerals for C2 and C4, including, respectively, the following notable macrominerals: potassium (508.08 and 555.77 mg/100 g), calcium (201.45 and 124.88 mg/100 g), magnesium (151.08 and 132.88 mg/100 g) and sodium (222.94 and 218.36 mg/100 g). Among the microminerals, the highest contents obtained were of iron and manganese in C2 (2.80 and 7.23 mg/100 g of cookies, respectively) and manganese (5.36 mg/100 g of cookies) in C4. Corroborating the results mentioned above, Msaddak et al. [40] noticed that the inclusion of cladode from prickly pear powder to cookies increased the amounts of potassium, magnesium, calcium, iron, and zinc.

Minerals are nutrients essential to metabolism and homeostasis in the human body, and deficiency of these bioactive constituents can result in common disorders and diseases [41]. Similarly, to fiber, reference values for their intake are periodically established [37].

Based on the consumption of a 50 g portion, equivalent to 3 cookies, and according to the DRIs, the calcium content in the cookies would account for 10.86 and 6.24% of the DRI when consuming C2 and C4 cookies, respectively, while control cookies C1 and C3 contained 4.52 and 4.93% of the DRI of calcium, respectively. Calcium is one of the most abundant minerals in the body and plays an important role in the structure and conservation of bones and teeth, muscle development, and regulation of the heart rate and blood pressure [42]. Concerning copper content, there was no difference between xiquexique cookies and their controls (p ≥ 0.05), and the 50 g cookies portion contained 13.89 (C1 and C2) and 23.33% (C3 and C4) of the DRI of copper. The presence of copper is relevant due to its antioxidant effects, its ability to stimulate the immune system, and its presence in the structure of many enzymes [41].

The iron content of C2 was 17.50% of the DRI of adults, while C1 provides only 15.88% of the DRI, despite wheat flour being supplemented with iron by law. Deficiency of this nutrient is associated with the development of iron deficiency anemia, decreased cognitive ability and behavioral changes in children [43, 44].

For magnesium, both xiquexique cookies (C2 and C4) presented high contents (p < 0.05), supplying 17.99 and 15.82% of the DRI, respectively, while their controls supplied only 12.33 (C1) and 7.75% (C3) of the DRI; therefore, we can say that xiquexique cookies are a source of magnesium and can be consumed for the purpose of supplying this nutrient. This mineral is related to several important metabolic reactions in the human body, as it is a cofactor in more than 300 enzymatic reactions [45].

The xiquexique cookies presented potassium contents that were two times higher than those of their respective controls (p < 0.05); 50 g of C2 and C4 supplied 5.41 and 5.91% of the DRI of potassium, while C1 and C3 supplied only 2.26 and 2.81% of the DRI, respectively. The addition of xiquexique flour in cookies can contribute to achieving the DRI of this mineral. Potassium is important for fluid balance, nerve transmission, muscle contraction, the maintenance of blood pressure and waste elimination [41].

The observed manganese values were particularly notable; the cookies supplied 157.50 (C2) and 116.52% (C4) of the adult RDI of manganese; therefore, xiquexique cookies contain more than enough manganese to meet the daily needs of adults. Manganese is an important trace element that is responsible for activating various enzymes in charge of scavenging free radicals, regulating glucose homeostasis, mobilizing calcium [46], increasing insulin secretion and improving glucose tolerance [47]. When compared to other cacti, Astello-Garcia et al. [48] found lower values of this mineral in *Opuntia* spp.

The consumption of 50 g of xiquexique cookies (3 units with approximately 16.6 g each) according to the DRI supplies 7.43 (C2) and 7.28% (C4) for sodium, 10.86 (C2) and 16.99% (C4) for phosphorus, 4.09 (C2) and 6.73% (C4) for zinc.

The values of minerals contained in the xiquexique cookies suggest that these cookies are a potential supply of these minerals. Even minerals that presented relatively low values, if combined with a balanced diet, can contribute to the provision of these nutrients. Therefore, it is verified that the xiquexique cookies present relevant contents of minerals important for human health and therefore could be additions to a daily diet.

### Color, texture and spread ratio

The cookies are pictorially represented in Fig 2 and the color characteristic for the same is represented in Table 4. It was observed that with the addition of xiquexique flour in the dough, the L* and a* parameters decreased (p < 0.05), causing the C2 and C4 cookies to become darker in color (L* values of 43.79 and 49.12, respectively) in comparison to C1 and C3 cookies (53.85 and 52.62, respectively); this can be clearly seen in Fig 2. However, the b* values of xiquexique cookies were higher than those of the controls (p < 0.05), indicating less red tonality and higher yellowness, probably due to the influence of the yellowish green color of xiquexique flour (Fig 1).

**Table 4:** Color, hardness and spread ratio.

Variable	C1	C2	C3	C4
L*	53.85^a^ ±0.02	43.79^b^ ±0.06	52.62^a^ ±0.01	49.12^b^ ±0.12
a*	15.27^a^ ±0.10	14.12^b^ ±0.05	14.62^a^ ±0.03	14.25^b^ ±0.01
b*	28.86^b^ ±0.04	35.21^a^ ±0.02	30.15^b^ ±0.01	35.58^a^ ±0.03
Fracture force (g)	844.13^b^ ±0.35	1864.93^a^ ±0.49	2439.63^b^ ±0.43	3052.18^a^ ±0.47
Fracture force (ΔE)		1020.80 ±0.14^A^		612.55 ±0.04^B^
Spread ratio	5.80^a^ ±0.02	3.04^b^ ±0.03	4.25^a^ ±0.01	3.62^b^ ±0.02
Diameter	5.13^a^ ±0.03	4.45^b^ ±0.06	4.75^a^ ±0.01	4.31^b^ ±0.03
Thickness	0.91^b^ ±0.06	1.51^a^ ±0.03	1.12^b^ ±0.03	1.22^a^ ±0.06

C1—wheat flour cookies (control1), C2–50% xiquexique flour (100 mesh) cookies, C3—whole wheat flour cookies (control2) and C4–50% whole xiquexique flour (28 mesh) cookies.

^a, b^Mean ± standard deviation with different lowercase letters on the same line differed significantly by Student’s t-test (p < 0.05) for C1 versus C2 and C3 versus C4.

^A, B^Mean ± standard deviation with different capital letters on the same row differed significantly by Student’s t-test (p < 0.05) for C2 versus C4.

C2 and C4 exhibited higher fracture force values than C1 and C3 (p < 0.05), however when the types of cookies were compared through the PF difference (ΔE), it was greater for conventional cookies. Giuberti et al. [38] observed the same behavior for facture force in alfalfa cookies. It is well recognized that the hardness of cookies is related to water-starch-protein interactions as a function of the composition of flour and the interplay among ingredients [49]. The higher fracture force values measured for C2 and C4 than for C1 and C3 could be related to the protein content, with higher protein levels contributing to the formation of a harder structure as a result of strong adherence between proteins and starch [50]. In xiquexique cookies, in addition to having the highest protein level (p < 0.05), the results also suggested that the presence of total fibers can influence texture values since higher levels of fiber can contribute to compacting the structure of the dough [51, 52]. Being a better result than a study of cookies with wheat malt that found less hardness which resulted in less acceptability and was related to the higher fiber content [53].

The spread ratio was lower in the cookies with xiquexique flour (C2 and C4) than in the control cookies (p < 0.05), as well as in bamboo cookies [16], chestnut cookies [54], and fiber-supplemented cookies [22]. Their values, as shown in Table 4, indicate that the spread ratio is affected by the competition of ingredients for available water. Ingredients such as insoluble fibers absorb water during mixing and reduce cookies spread [54]. Thus, the lowest values of the spread ratio in xiquexique cookies can be explained by the higher insoluble fiber content than that of the control cookies.

### Sensorial evaluation

The sensorial tests were carried out using 123 voluntary individuals (aged 18–60 years). The results of the acceptance and preference of the cookies are shown in Table 5. All cookies scored between 6.1 and 7.0 for all sensory acceptance attributes, which corresponds to a range of "like slightly" to "like moderately". C2 did not differ in appearance, color and texture when compared to C1 (p ≥ 0.05), while C4 did not differ in appearance, flavor, texture or general perception when compared to C3 (p ≥ 0.05).

**Table 5:** Acceptance scores and preference of the cookies.

Variable	C1	C2	C3	C4
Appearance	7.00^a^ ±1.10	7.00^a^ ±1.30	6.90^a^ ±1.10	6.70^a^ ±1.20
Color	7.00^a^ ±1.20	7.00^a^ ±1.30	7.10^a^ ±1.10	6.80^b^ ±1.30
Flavor	7.00^a^ ±1.10	6.50^b^ ±1.30	6.70^a^ ±1.10	6.80^a^ ±1.20
Texture	7.30^a^ ±1.30	6.90^a^ ±1.30	6.10^a^ ±1.60	6.00^a^ ±1.60
Taste	7.30^a^ ±1.00	6.20^b^ ±1.60	6.90^a^ ±1.10	6.60^b^ ±1.40
General perception	7.30^a^ ±1.10	6.40^b^ ±1.60	6.80^a^ ±1.20	6.50^a^ ±1.40

C1—wheat flour cookies (control1), C2–50% xiquexique flour (100 mesh) cookies, C3—whole wheat flour cookies (control2) and C4–50% whole xiquexique flour (28 mesh) cookies.

^a, b^Mean ± standard deviation with different letters on the same row differed significantly by Student’s t-test (p < 0.05) for C1 versus C2 and C3 versus C4.

The general perception for C3 and C4 was the same (p ≥ 0.05), suggesting that the addition of whole xiquexique flour did not influence this parameter. Moro et al. [55] observed similar behavior and claimed that this indicates relatively high approval of ingredients with health benefits, even when possibly affecting the sensory characteristics of the products.

**Fig 3:**
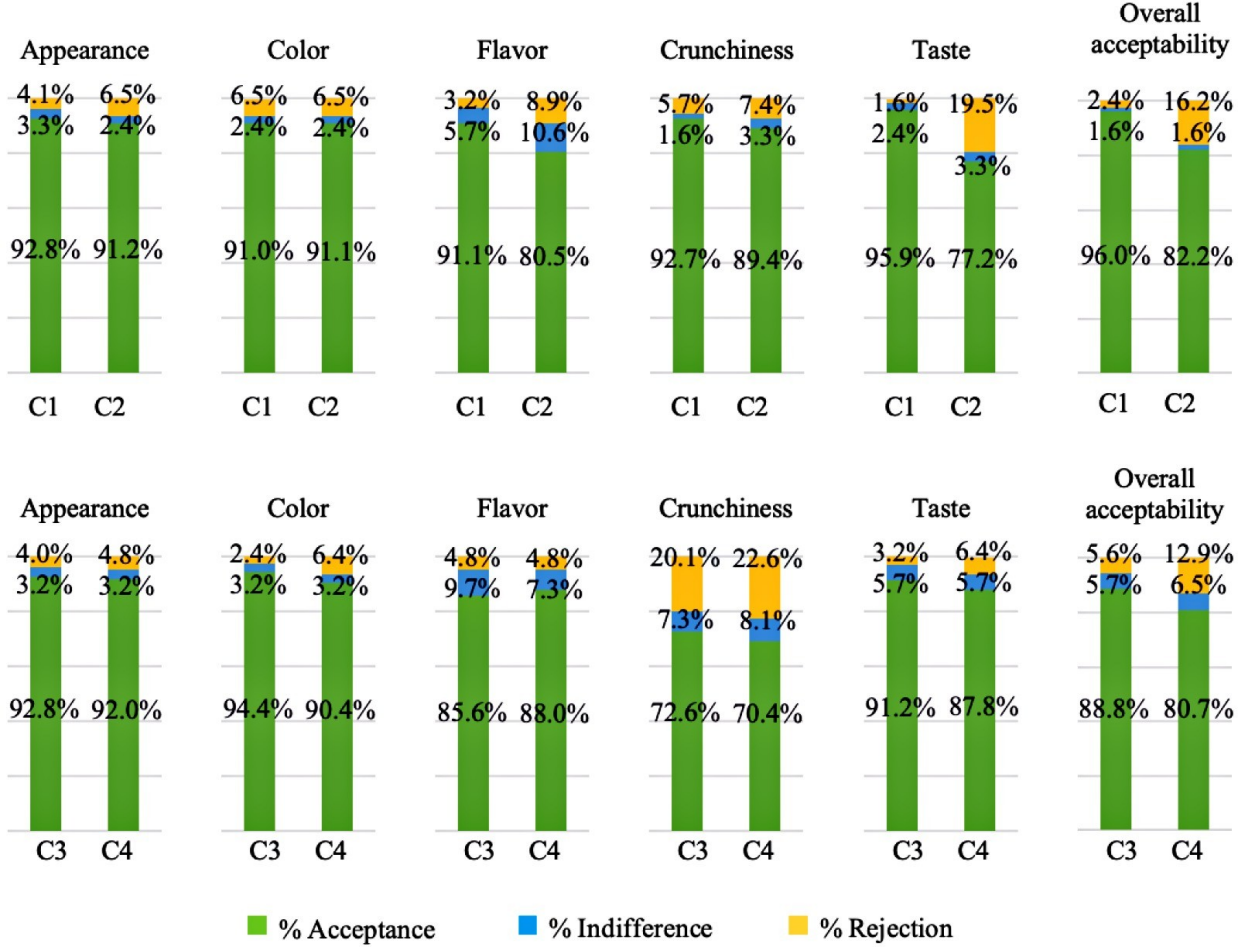
Acceptability of the appearance, color, flavor, crunchiness, taste and overall acceptability of the cookies. Acceptance, indifference, and rejection percentages for the acceptability of the appearance, color, flavor, crunchiness, taste and overall acceptability of the cookies. C1—wheat flour cookies (control1), C2–50% xiquexique flour (100 mesh) cookies, C3—whole wheat flour cookies (control2) and C4–50% whole xiquexique flour (28 mesh) cookies.

The percentages of acceptance (corresponding to values 9 to 6), indifference (value 5) and rejection (values 4 to 1) are presented in Fig 3. These values are associated with the samples by means of the hedonic scale used for the evaluation of the appearance, color, flavor, crunchiness, taste and overall acceptance. All evaluated parameters were well accepted by most of the consumers, achieving more than 70% acceptance for all evaluated attributes. This greater acceptance represents an advance in the sensory acceptance of unconventional flour products by consumers.

**Fig 4:**
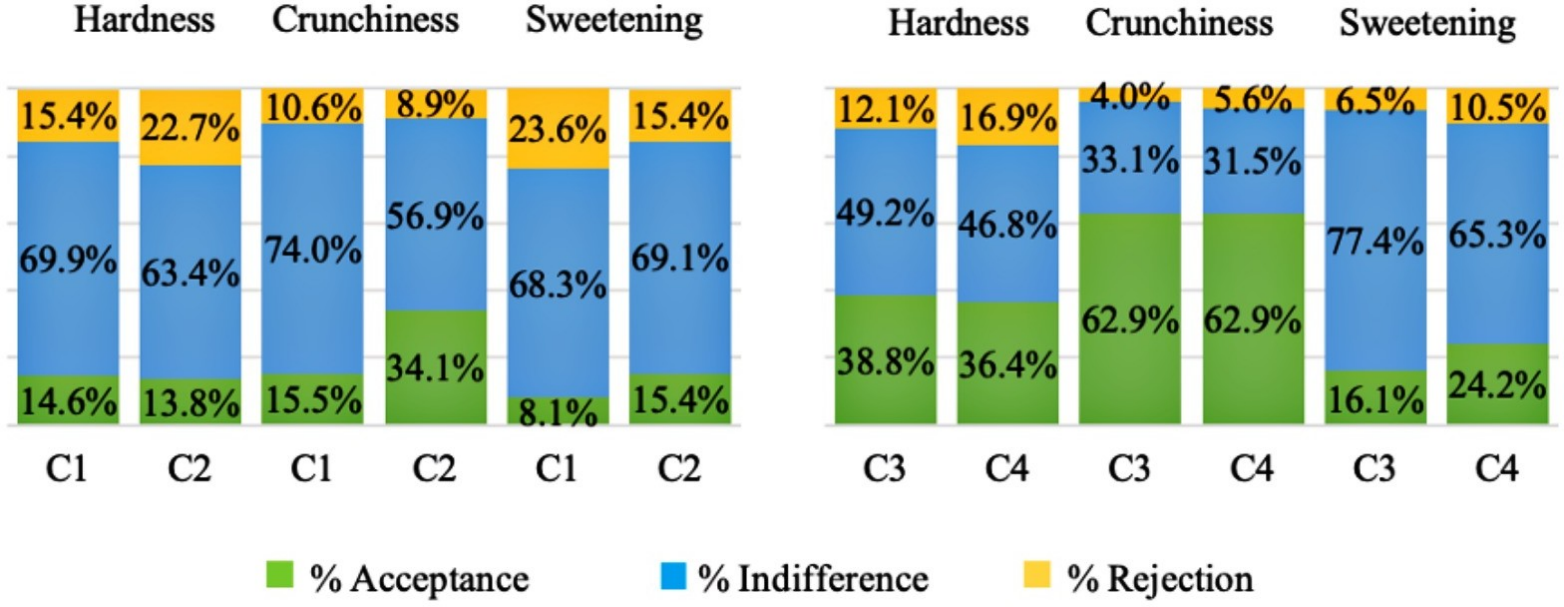
Hardness, crunching and sweetness of the cookies. Ideal rating percentages above and below ideal hardness when biting and crunching and sweetness of the cookies. C1—wheat flour cookies (control1), C2–50% xiquexique flour (100 mesh) cookies, C3—whole wheat flour cookies (control2) and C4–50% whole xiquexique flour (28 mesh) cookies. *Perceived by more than 20% of consumers, with a significant decrease of at least 1 point in the average overall acceptability.

In Fig 4, we can observe the percentages of classification within the above ideal (values 5 and 4), ideal (value 3) and below ideal (values 2 and 1) acceptance levels, from hardness when biting, crunching and sweetness, in addition to the results of the analysis of penalties. This analysis calculates the difference between the means of the groups and determines the optimal attributes. The means of the groups with more than 20% of responses above or below the ideal for a given attribute may be an indication that the product requires improvement in terms of this attribute.

It was observed that 34.1% of the consumers judged the crunchiness of C2 to be less intense than ideal, which caused a significant reduction in general perception. In addition, for 22.7% of the consumers, C2 was evaluated as harder than ideal but had no impact on the average of general perception (Table 5). Likewise, C1 was considered sweeter than ideal by 23.6% of consumers; however, it did not affect the average overall acceptability. Relative whole cookies, both C3 and C4, were considered to be less harsh than ideal by 38.8 and 36.4% of the consumers, respectively. In addition, 62.9% of consumers judged both samples to be less crunchy than ideal. The sweetness of C4 was considered less intense than ideal by 24.2% of the consumers.

**Fig 5:**
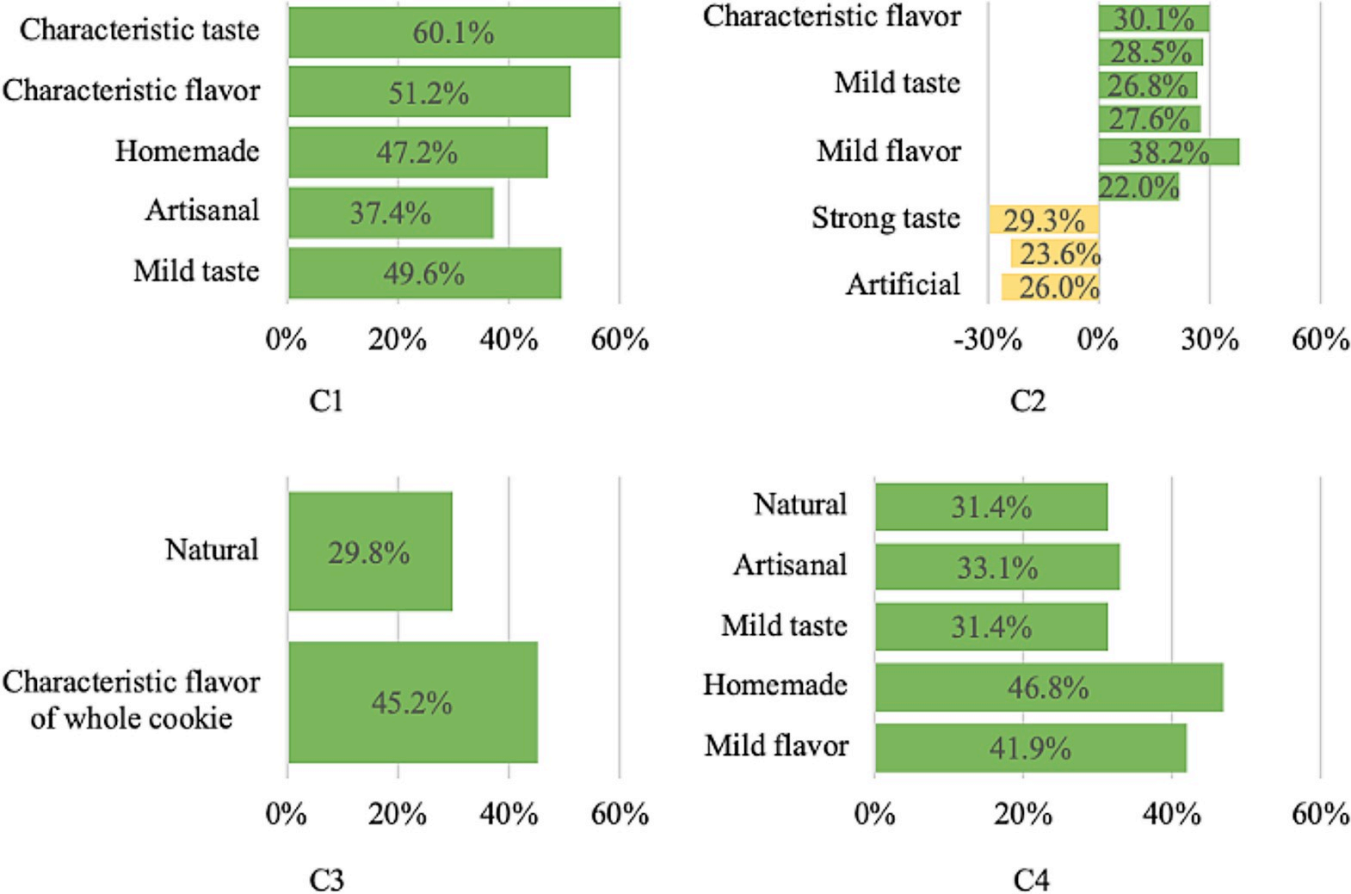
Attributes with a significant effect on the overall acceptability of the cookies. C1—wheat flour cookies (control1), C2–50% xiquexique flour (100 mesh) cookies, C3—whole wheat flour cookies (control2) and C4–50% whole xiquexique flour (28 mesh) cookies.

The differences in the consumers’ perception of cookies can be observed by the CATA test in Fig 5. C1 was described as having a characteristic taste (60.1%), having a characteristic flavor (51.2%), being homemade (47.2%), being artisanal (37.4%) and having a mild taste (49.6%) by consumers, with an increase in the overall acceptability average of between 0.4 and 0.8 points.

C2 was described as having a characteristic flavor (30.1%), having a characteristic taste (28.5%), having a mild taste (26.8%), being artisanal (27.6%), having a mild flavor (38.2%), being natural (22.0%), having a strong taste (29.3%), having an uncharacteristic taste (23.6%) and being artificial (26.0%) by consumers, with an average positive impact on the overall acceptability of 0.7 to 1.5 points, in addition to being described as artificial and noncharacteristic and as having strong flavor by 23.6 and 30.1% of consumers, with a reduction of 0.9 to 1.2 points in overall acceptability.

Concerning C3 was described as being natural (29.8%) and having a characteristic whole cookies aroma (45.2%), with an increase in the overall acceptability average between 0.9 and 0.5 points. C4 was described as being natural (31.4%), being artisanal (33.1%), having a mild taste (31.4%), being homemade (46.8%) and having a mild flavor (41.9%) by consumers, which had a positive impact on the overall acceptability average of 0.5 to 0.9 points. C3 and C4 samples were described by more than 20% of consumers as having attributes that caused a reduction in the overall acceptability average.

**Fig 6:**
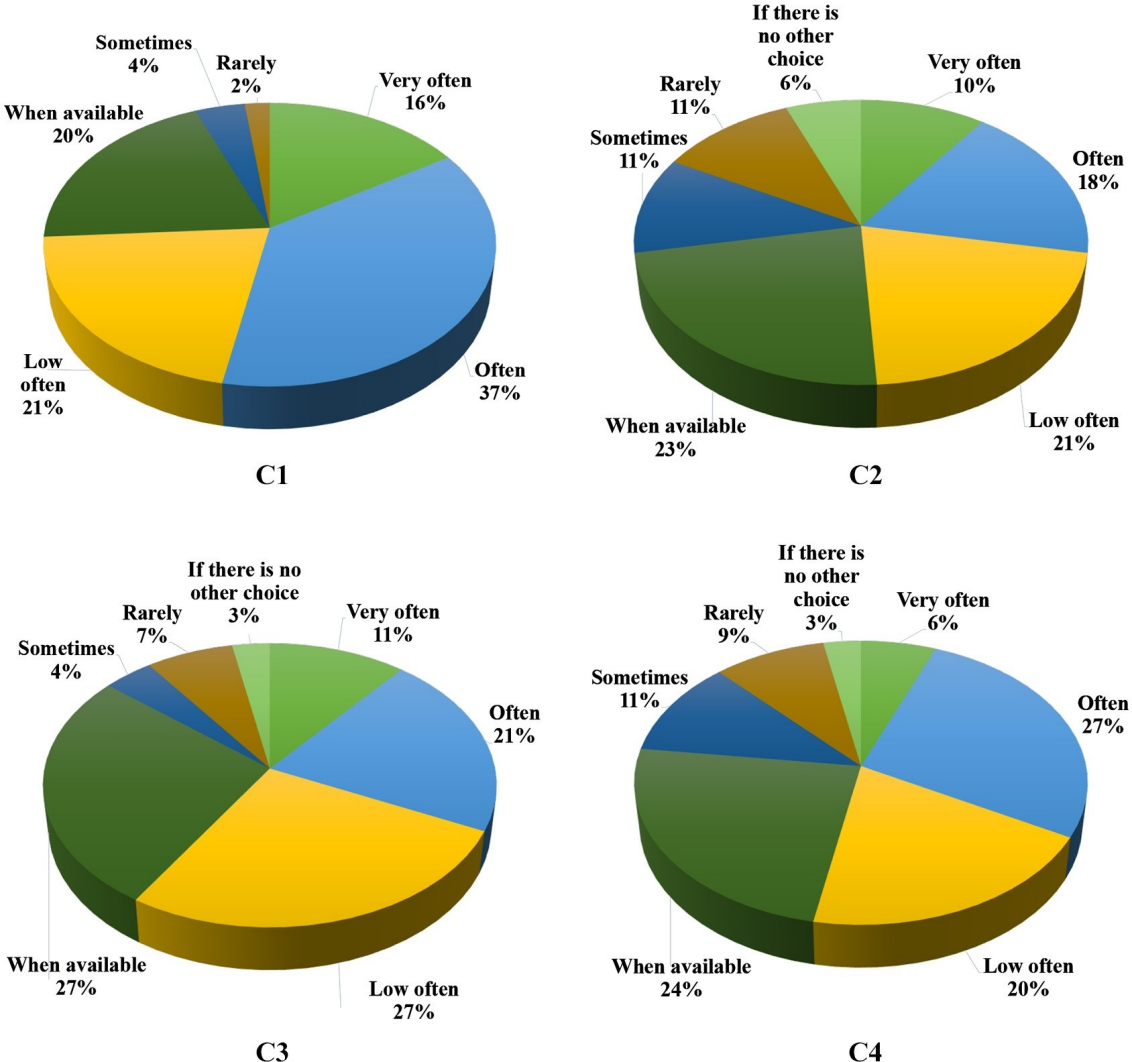
Intention of consumption frequency of the cookies. C1—wheat flour cookies (control1), C2–50% xiquexique flour (100 mesh) cookies, C3—whole wheat flour cookies (control2) and C4–50% whole xiquexique flour (28 mesh) cookies.

The consumer intent results are presented in Fig 6. C2 and C1 were scored from 1 to 7, corresponding to “if there is no choice", "rarely", "sometimes", "when available", "not very often", "often" and "very often”. All the formulations were not rejected by consumers; the cookies with the addition of xiquexique flour exhibited results indicating that only 6 and 3% of consumers would consume the cookies C2 and C4, respectively, “if there is no other choice”, representing an advance in the sensory acceptance of whole-grain products, which are known to alter the conventional flavor to the consumer, indicating enhanced approval of ingredients with health benefits, even when possibly affecting the sensory characteristics of the products [55]. Thus, we can say that the xiquexique flour (tamized at 100 and 28 mesh) present a potential for the development of baked goods such as cookies.

## Counterpoints that justify the importance of replacing wheat flour with cactus flour in the processing of food products

Wheat is a food matrix for global consumption, but its cultivation in the quantities necessary to reach consumer demand is not viable due to the damage that its cultivation brings to the soil as impoverishment and desertification. In addition, the food industry is constantly developing strategies to reduce manufacturing increase profitability and improve the nutritional quality of products. In this matter, the incorporation of alternative ingredients in wheat-based food products represents a promising strategy to meet the demand for nutritious, sustainable and agro-ecological products, meeting consumer demands [56].

Within this context, the replacement of wheat flour by alternative flours of cacti [35, 36], specifically that of xiquexique, is of paramount importance both for their interesting composition and in a wider perspective of agroecology, in view of climate change and all the environmental challenges facing the planet [57]. When talking about unconventional plants (UCP), such as cacti, the lowest production cost is inseparable when compared to traditional monocultures, given that there is no use of agrochemicals as well as all cultivation management is simplified and we still have the lowest land cost, as it does not wear out as in traditional monoculture and so the need for soil is much less; furthermore, there is also no need for burning in soils which of the factors mentioned above still pollutes the air. We also have less fossil fuel consumption considering that it can be produced in many arid ecosystems which are not feasible to produce wheat without excessive expenditure on irrigation and other factors.

In this study, the initial hypothesis of the viability of using xiquexique flour as a partial gluten substitute was verified by the acceptability of the cookies and by the verified textural properties. In addition, there is a greater richness in fibers and minerals in cookies made with partial replacement of wheat flour by xiquexique flour. It is noteworthy that this substitution did not compromise technological properties, such as the kneading ability, nor the acceptance of most of the evaluated sensory attributes, reinforcing the importance of this study. So, the partial substitution of wheat flour with xiquexique flour to produce cookies is a promising strategy in the manufacturing of bakery products. The possibility of testing the xiquexique flour for cake preparation could be another interesting field of application.

## Conclusions

This is the first evidence of the use of xiquexique flour in the processing of cookies and suggests it as an excellent ingredient for production this type of product. The cookies with added xiquexique flour obtained the highest values of darkness, hardness, ash, protein and resistant starch contents, as well as low water activity values, moisture contents and spread ratios when compared to their respective controls. Xiquexique cookies can be considered as an innovative product for the functional food industry, especially for its bioactive and nutritional characteristics, being excellent sources of fibers and minerals, essential nutrients for the general population. Our study shows that cookies added from finely xiquexique flour (tamized at 100 mesh) and the xiquexique flour (tamized at 28 mesh) were well accepted by consumers. The CATA method added another dimension (technological attributes perception) to describe products. The results of this method can be used for the adjustment of cookies recipes and the development of optimal cookies and other baked goods with partial replacement of wheat flour.

The authors declare the Impact of the Novelty, considering all the aspects already reported and reinforcing that the present invention (cookies enriched with xiquexique flour) has unique characteristics, being a potential alternative for nutritionally rich, tasty, safe and practical food; and thus represents a new form of economic use of this plant species, especially in relation to sustainability.

## Supporting information

S1 Table Characterization of the xiquexique flour. F1—Xiquexique flour tamized at 100 mesh; F2—Xiquexique flour tamized at 28 mesh—Flours chosen to process cookies C2 and C4, respectively. ^a, b^Media ± standard deviation with different letters on the same line differed by Student’s t-test (p < 0.05), between treatments.
(DOCX)

S2 Table Minerals profile in mg/100 g of xiquexique flour. F1—Xiquexique flour tamized at 100 mesh; F2—Xiquexique flour tamized at 28 mesh—Flours chosen to process cookies C2 and C4, respectively. ^a, b^Media ± standard deviation with different letters on the same line differed by Student’s t-test (p < 0.05), between treatments. *Based in Institute of Medicine. Dietary Reference Intakes, Washington D. C., National Academy Press; 2003 (1997–2005). Based on a 70 kg man, 31–50 years old [34]. (1) Adequate Intake; (2) Recommended Dietary Allowances.
(DOCX)
