# Supplementary material for: Nutritional, physicochemical and sensorial acceptance of functional cookies enriched with xiquexique (Pilosocereus gounellei) flour
Source: PLoS One. 2021 Aug 10;16(8):e0255287. doi: 10.1371/journal.pone.0255287 (PMC8354474; doi:10.1371/journal.pone.0255287)
Supplement: S1 Table — F1—Xiquexique flour tamized at 100 mesh; F2—Xiquexique flour tamized at 28 mesh—Flours chosen to process cookies C2 and C4, respectively. a, bMedia ± standard deviation with different letters on the same line differed by Student’s t-test (p < 0.05), between treatments. (DOCX) [file pone.0255287.s001.docx]

**S1 Table**

Characterization of the xiquexique flour.

| Variable | F1 | F2 |
| --- | --- | --- |
| Starch (%) | 61.31^a^ ±0.20 | 46.23^b^ ±0.09 |
| Resistent starch (%) | 4.40^b^ ±0.02 | 5.65^a^ ±0.01 |
| Fibers total (%) | 16.59^b^ ±0.09 | 29.81^a^ ±0.21 |
| Fibers insoluble (%) | 12,67^b^ ±0.09 | 24,16^a^ ±0.21 |
| Fibers soluble (%) | 3.92^b^ ±0.05 | 5.65^a^ ±0.07 |

F1 - Xiquexique flour tamized at 100 mesh; F2 - Xiquexique flour tamized at 28 mesh - Flours chosen to process cookies C2 and C4, respectively.

^a-b^Media ± standard deviation with different letters on the same line differed by Student's t-test (p <0.05), between treatments.
