# Supplementary material for: Nutritional, physicochemical and sensorial acceptance of functional cookies enriched with xiquexique (Pilosocereus gounellei) flour
Source: PLoS One. 2021 Aug 10;16(8):e0255287. doi: 10.1371/journal.pone.0255287 (PMC8354474; doi:10.1371/journal.pone.0255287)
Supplement: S2 Table — F1—Xiquexique flour tamized at 100 mesh; F2—Xiquexique flour tamized at 28 mesh—Flours chosen to process cookies C2 and C4, respectively. a, bMedia ± standard deviation with different letters on the same line differed by Student’s t-test (p < 0.05), between treatments. *Based in Institute of Medicine. Dietary Reference Intakes, Washington D. C., National Academy Press; 2003 (1997–2005). Based on a 70 kg man, 31–50 years old [34]. (1) Adequate Intake; (2) Recommended Dietary Allowances. (DOCX) [file pone.0255287.s002.docx]

**S2 Table**

Minerals profile in mg/100 g of xiquexique flour.

| Elements | F1 | F2 | *Recommendation (mg) |
| --- | --- | --- | --- |
| K | 264.57^b^ ±3.53 | 555.77^a^ ±1.93 | 4700^(1)^ |
| Ca | 98.63^b^ ±0.89 | 124.88^a^ ±0.45 | 1000^(1)^ |
| P | 246.50^a^ ±0.82 | 237.81^b^ ±0.88 | 700^(2)^ |
| Mg | 65.14^b^ ±1.53 | 132.88^a^ ±2.12 | 420^(2)^ |
| Na | 173.95^b^ ±3.01 | 218.36^a^ ±2.98 | 1500^(1)^ |
| Cu | 0.44^a^ ±0.01 | 0.42^a^ ±0.01 | 0.9^(2)^ |
| Fe | 3.19^a^ ±0.01 | 2.87^b^ ±0.02 | 8^(1)^ |
| Mn | 1.69^b^ ±0.08 | 5.36^a^ ±0.17 | 2.3^(1)^ |
| Zn | 1.59^a^ ±0.03 | 1.48^b^ ±0.01 | 11^(2)^ |

F1 - Xiquexique flour tamized at 100 mesh; F2 - Xiquexique flour tamized at 28 mesh - Flours chosen to process cookies C2 and C4, respectively.

^a-b^Media ± standard deviation with different letters on the same line differed by Student's t-test (p <0.05), between treatments.

*Based in Institute of Medicine. Dietary Reference Intakes, Washington D. C., National Academy Press; 2003 (1997-2005). Based on a 70 kg man, 31-50 years old [34]. (1)Adequate Intake; (2)Recommended Dietary Allowances.
